# Interaction of the European genotype porcine reproductive and respiratory syndrome virus (PRRSV) with sialoadhesin (CD169/Siglec-1) inhibits alveolar macrophage phagocytosis

**DOI:** 10.1186/1297-9716-43-47

**Published:** 2012-05-25

**Authors:** Miet I De Baere, Hanne Van Gorp, Peter L Delputte, Hans J Nauwynck

**Affiliations:** 1 Laboratory of Virology, Department of Virology, Parasitology and Immunology, Faculty of Veterinary Medicine, Ghent University, Salisburylaan 133, 9820, Merelbeke, Belgium; 2 PROVAXS, Ghent University-Ghent University Hospital, De Pintelaan 185, 9000, Ghent, Belgium

## Abstract

Porcine reproductive and respiratory syndrome virus (PRRSV) is an arterivirus that shows a restricted in vivo tropism for subsets of porcine macrophages, with alveolar macrophages being major target cells. The virus is associated with respiratory problems in pigs of all ages and is commonly isolated on farms with porcine respiratory disease complex (PRDC). Due to virus-induced macrophage death early in infection, PRRSV hampers the innate defence against pathogens in the lungs. In addition, the virus might also directly affect the antimicrobial functions of macrophages. This study examined whether interaction of European genotype PRRSV with primary alveolar macrophages (PAM) affects their phagocytic capacity. Inoculation of macrophages with both subtype I PRRSV (LV) and subtype III PRRSV (Lena) showed that the virus inhibits PAM phagocytosis. Similar results were obtained using inactivated PRRSV (LV), showing that initial interaction of the virion with the cell is sufficient to reduce phagocytosis, and that no productive infection is required. When macrophages were incubated with sialoadhesin- (Sn) or CD163-specific antibodies, two entry mediators of the virus, only Sn-specific antibodies downregulated the phagocytic capacity of PAM, indicating that interaction with Sn, but not CD163, mediates the inhibitory effect of PRRSV on phagocytosis. In conclusion, this study shows that European genotype PRRSV inhibits PAM phagocytosis in vitro, through the interaction with its internalization receptor Sn. If similar events occur in vivo, this interaction may be important in the development of PRDC, as often seen in the field.

## Introduction

Porcine reproductive and respiratory syndrome virus (PRRSV) is the causative agent of porcine reproductive and respiratory syndrome (PRRS), which has become one of the most important diseases affecting swine industry worldwide. PRRS is characterized by reproductive failure in sows and gilts and respiratory problems in pigs of all ages [1,2]. The annual cost of this disease is estimated at 664 million dollars in the USA alone [3], representing over one-third of the total losses in swine industry attributed to infectious diseases [4]. PRRSV is a small, enveloped, positive-stranded RNA virus that belongs to the genus *Arterivirus*, which is classified within the family of the *Arteriviridae*, in the order *Nidovirales*[5]. Two distantly related genotypes exist: the European genotype (type I) and the North American genotype (type II) [6,7]. Within each genotype a high degree of genetic variability has been described [8,9], which led to the recent proposal to subdivide the European genotype into different subtypes [10,11]. Different PRRSV isolates also display a remarkable antigenic and pathogenic variability. Their virulence ranges from apathogenic or moderately virulent, to highly pathogenic, like the recently emerging type II isolates described in China [12] and the type I subtype III isolate PRRSV (Lena), originating from Belarus [13].

PRRSV shows a restricted in vivo host tropism, infecting pigs only, as well as a restricted cell tropism for cells of the monocyte/macrophage lineage. The latter is reflected by the specific infection of subsets of macrophages that are mainly present in lungs, lymphoid tissues and placenta [14,15], with alveolar macrophages being major target cells [16]. The extensive PRRSV replication in the lungs results in lysis or apoptosis of infected cells and apoptosis of uninfected bystander cells [17-20]. In previous studies [21,22], a decrease in the number of alveolar macrophages was found up to 9 days post infection, with a maximum decrease of 35–40%, followed by a marked increase up to 52 days post infection, resulting from replacement of these cells by infiltrating monocytes, which are known to differentiate into macrophages. Since alveolar macrophages are the predominant cells involved in the innate defence in the lungs [23], PRRSV infection impacts the respiratory immune system of the pig early in infection. The virus is associated with respiratory problems in pigs of all ages, is known to predispose pigs to secondary infections, and is commonly isolated on farms with porcine respiratory disease complex (PRDC) [24].

PRRSV shows a tropism for specific subsets of differentiated macrophages [14], which is conferred by the presence of certain entry mediators. As reviewed by Van Breedam et al. [25], three entry mediators are known to be involved in the entry pathway of PRRSV in macrophages: 1) heparan sulphate glycosaminoglycans serve as PRRSV attachment factors involved in the initial binding and concentration of the virus on the macrophage surface; 2) the siglec sialoadhesin (Sn) interacts with sialic acids present on the virus, which leads to a more stable binding, and results in internalization of the virus together with its receptor; 3) scavenger receptor CD163 is implicated in virus uncoating and genome release. It is established that Sn functions as a clathrin-dependent endocytic receptor that can be targeted by Sn-specific immunotoxins to kill macrophages and by Sn-specific antigen immunoconjugates as a vaccination strategy improving antibody responses [26]. Also, in a recent study it was shown that antibody binding to porcine Sn causes a decrease in phagocytic capacity of primary alveolar macrophages (PAM) [27]. It was suggested that the interaction of other ligands with Sn might also result in an impaired phagocytic capacity of PAM. Considering the reported increase in secondary bacterial infections following PRRSV infection, the downregulating effect of antibody binding to Sn on macrophage phagocytosis and the fact that PRRSV interacts with Sn, we wondered whether PRRSV binding to Sn might likewise result in a decreased phagocytic capacity of PAM. Therefore, this study aimed to assess whether the interaction of European genotype PRRSV with PAM affects their phagocytic capacity, and whether interaction with its entry mediators Sn and/or CD163 mediates the inhibitory effect of PRRSV on PAM phagocytosis.

## Materials and methods

### Cells

PAM were obtained from 4- to 6-week-old conventional Belgian Landrace pigs from a PRRSV-negative herd as previously described [2]. PAM and Marc-145 cells were cultivated as described by Van Gorp et al. [28].

### Viruses

Marc-145-grown virus: a 4^th^ passage was used for the following PRRSV strains: 1) genotype I, subtype I prototype PRRSV strain, Lelystad virus (LV), that was first passaged on macrophages for 12 passages [2]; 2) genotype I, subtype III prototype PRRSV strain Lena, that was adapted on Marc-145 cells as described [13]. For concentration and inactivation, a 5^th^ passage of PRRSV (LV), that was first passaged on macrophages for 12 passages, was used. Macrophage-grown virus: a 13^th^ passage of PRRSV (LV) and a 4^th^ passage of PRRSV (Lena) was used. For concentration, a 14^th^ passage of PRRSV (LV) and a 4^th^ passage of PRRSV (Lena), were used. Virus titrations and calculations of the virus titres (TCID_50_ values) were performed as described before [28]. For each experiment, virus titrations were performed on macrophages from the same batch as the batch used in the respective experiment. Virus was concentrated from the supernatant after filtration through a 0.45 μm filter (Sarstedt, Nümbrecht, Germany) as previously described [29]. Virus inactivation through treatment with binary ethylenimine (BEI; Sigma-Aldrich, St. Louis, MO, USA) or with ultraviolet (UV) radiation was performed and verified as described before [29], always confirming complete inactivation.

### Antibodies

The IgG_1_ porcine Sn-specific mouse monoclonal antibody (mAb) 41D3 was used as a ligand to bind Sn [30,31]. The IgG_1_ porcine CD163-specific mouse mAb 2A10 (AbD Serotec, Kidlington, UK; MCA2311) or a human CD163-specific goat polyclonal antibody (pAb) (R&D Systems, Minneapolis, MN, USA), which is known to cross-react with porcine Sn, were used as ligands to bind CD163 [28,32,33]. Isotype-matched irrelevant mAb 13D12, directed against pseudorabies virus glycoprotein gD [34], and purified goat antibodies were used as controls. MAb 41D3 and 13D12 were purified using protein G column chromatography (GE Healthcare, VWR, Leuven, Belgium) following the manufacturers’ instructions. PRRSV virions were visualized via the nucleocapsid protein-specific mAb 13E2 [35].

### Phagocytosis assays

To assess the effect of PRRSV or Sn- and CD163-specific antibodies on PAM phagocytosis, macrophages were inoculated with PRRSV or antibodies 24 h post seeding and further incubated for 1 h, after which a phagocytosis assay was performed. In a first experiment PAM were inoculated with macrophage- or Marc-145-grown virus at a multiplicity of infection (moi) of 0.3, 1, 3, or 5 for PRRSV (LV) or an moi of 0.3, 1, 3, 5, 10 or 30 for PRRSV (Lena). The moi was calculated as the ratio between the TCID_50_ value and the number of cells. As a control, PAM were mock-infected with macrophage- or Marc-145-supernatant of cells that were cultured for 5 days. In a second experiment PAM were inoculated with an moi of 0.1, 0.3, 1, 3, 10 or 30 of concentrated Marc-145-grown PRRSV (LV) or equal amounts of concentrated and inactivated Marc-145-grown PRRSV (LV). As a control, PAM were mock-infected with RPMI containing 10% PBS, which is the medium in which the concentrated virus is dissolved (and inactivated). As a control for the effect of the BEI-treatment of the virus on PAM phagocytosis, cells were mock-infected with BEI-treated RPMI containing 10% PBS, showing no effect on phagocytosis. In a third experiment PAM were inoculated with 0.15, 0.5, 1.5, 5, 15, 50 μg/mL mAb 41D3, 2A10 or 13D12 or the CD163-specific pAb. As a control, PAM were inoculated with PBS in the absence of mAbs. Also, for every experiment a phagocytosis assay was performed at 4°C, following mock-infection, to determine the number of beads bound to the cell surface. Phagocytosis assays using fluorescent beads were performed in suspension and were analyzed by flow cytometry following a propidium iodide (Molecular Probes, Eugene, OR, USA) live-dead staining as described before [27]. Data were gated on FSC and SSC to remove debris from the analysis and on FL-2 to exclude non-viable cells from the analysis. The percentage of viable PAM phagocytosing beads and their MFI (median fluorescence intensity), which is a measure for the number of beads present per cell, was determined. Confocal microscopical pictures were generated as described before [27], including a staining for PRRSV particles using 13E2 as primary antibody, followed by goat-anti-mouse-FITC.

### Analysis of virus internalization in PAM

Twenty-four hours post seeding, adherent cells were washed once with RPMI-1640 (Gibco, Invitrogen, Carlsbad, CA, USA) followed by inoculation with different PRRSV strains at an moi of 1. Inoculated cells were incubated for 1 h at 37°C in the presence of the virus. After virus removal, cells were washed 3 times to remove unbound virions and an immunofluorescence staining was performed as described before [29,36]. Virus internalization in PAM was analyzed as previously described [29,36], analyzing at least 20 cells per sample.

### Statistics

For each experiment, PAM and virus from the same batch were used and each experiment was independently performed at least three times. Results are expressed as mean ± error of the mean (SEM). Statistical data analysis was performed using the GraphPad PRISM software package (v 5.0; La Jolla, CA, USA) using an unpaired *t*-test (analysis of the number of virions for macrophage- and Marc-145-grown PRRSV strains) or a paired *t*-test for all other analyses. *P* values < 0.05 were considered significant (* *p* < 0.05; ** *p* < 0.01; *** *p* < 0.001).

## Results

### European genotype PRRSV downregulates the phagocytic capacity of PAM, depending on the number of internalized virions

#### European genotype PRRSV downregulates the phagocytic capacity of PAM

**Figure 1:**
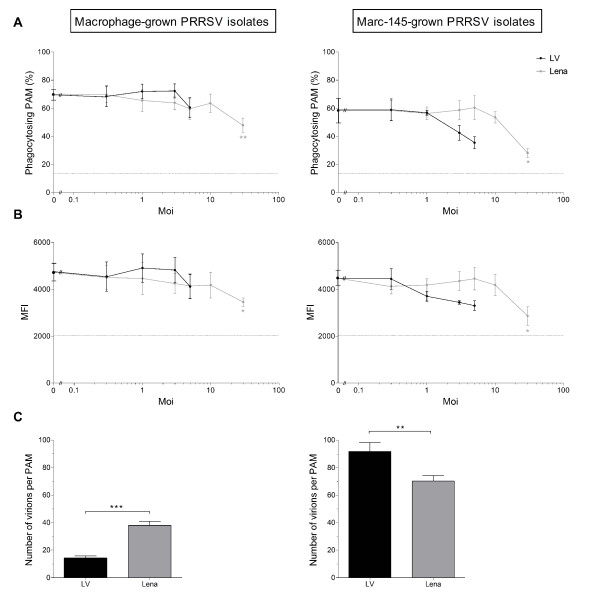
**European genotype PRRSV downregulates PAM phagocytic capacity, depending on the number of internalized virions.** Flow cytometric analysis of the effect of European genotype PRRSV entry on PAM phagocytosis and confocal microscopical analysis of the number of internalized PRRSV virions per PAM for Marc-145- and macrophage-grown PRRSV (LV) (Black circle) or PRRSV (Lena) (Grey circle): **(A–B)** The effect of both PRRSV strains on phagocytosis of beads by viable macrophages, expressed as the percentage of viable cells that have beads associated with them **(A)** or the MFI per viable cell **(B)**, which is a measure for the amount of beads per cell. PAM were inoculated with both PRRSV strains at different moi as indicated and infection was allowed for 1 h, after which a phagocytosis assay was performed. Mock infected cells were included as a positive control. In addition, a phagocytosis assay was performed at 4°C (dotted line) after mock infection, to assess the percentage of macrophages that have beads bound to their surface and their MFI. Data represent the mean ± SEM of 3 independent experiments; **(C)** PAM were inoculated with both PRRSV strains (moi = 1) and further incubated for 1 h, after which the number of virions per PAM was determined by an immunofluorescence staining. At least 20 cells were analyzed per sample. Data represent the mean ± SEM of 1 representative experiment. *p < 0.05; **p < 0.01; ***p < 0.001.

To study whether European genotype PRRSV has an impact on phagocytosis by PAM, cells were inoculated with PRRSV (LV) and PRRSV (Lena) at various moi and further incubated at 37°C for 1 h, after which a phagocytosis assay was performed. Only viable macrophages were included in the flow cytometric analysis. Interaction of both PRRSV strains with PAM causes a reduction in phagocytosis by PAM (Figure 1A and 1B). For PRRSV (LV) this inhibition is observed, even though not statistically significant (*p* = 0.11 for macrophage-grown and *p* = 0.06 for Marc-145-grown PRRSV (LV) at an moi of 5). The analysis shows that both the percentage of macrophages phagocytosing beads (Figure 1A) and the number of beads phagocytosed per macrophage, expressed as MFI (Figure 1B), are downregulated. This observation is independent from the cell type on which the virus is grown, since both Marc-145- and macrophage-grown PRRSV strains are able to inhibit the phagocytic capacity of PAM.

#### The amount of internalized PRRSV virions corresponds with the degree of European genotype PRRSV inhibition of PAM phagocytic capacity

Since the European genotype PRRSV inoculums used in the previous experiment contain both infectious and non-infectious virions and since the multiplicity of infection, which was calculated as the ratio between the TCID_50_ value and the number of cells, does not take into account the absolute number of virions entering a cell, we wondered whether a difference in the number of internalized virions corresponds with a difference in inhibition of PAM phagocytosis. To investigate this, an internalization experiment was performed (Figure 1C). Internalized or surface bound virions were never observed after mock infection. Interestingly, a higher number of bound and internalized virions corresponds with a higher reduction in phagocytosis for macrophage- and Marc-145-grown PRRSV (LV) and PRRSV (Lena) (Figure 1A and 1B). For macrophage-grown strains, PRRSV (LV) has a lower number of bound and internalized virions compared to PRRSV (Lena). This results in an inhibition of phagocytosis only starting from an moi of 5, whereas PRRSV (Lena) inhibits phagocytosis starting from an moi of 1. On the other hand, for Marc-145-grown strains, PRRSV (LV) has a higher number of bound and internalized virions compared to PRRSV (Lena) and inhibits phagocytosis starting from an moi of 3, compared to an moi of 10 for PRRSV (Lena).

As a control, to assess whether administering higher numbers of virions leads to a higher reduction in phagocytosis, macrophage-grown PRRSV (LV) and PRRSV (Lena) were concentrated. PAM were inoculated with concentrated macrophage-grown PRRSV (LV) (moi = 10) or PRRSV (Lena) (moi = 100) and further incubated at 37°C for 1 h, after which a phagocytosis assay was performed. Indeed, phagocytosis of PAM infected with PRRSV (LV) (moi = 10) was further reduced down to 42%, compared to 68.3% for mock-infected PAM. Phagocytosis of PAM infected with PRRSV (Lena) (moi = 100) was reduced down to 9.4%, which equals the percentage observed when the assay was performed at 4°C (10%), when active intracellular uptake is blocked, yet passive surface adsorption of beads proceeds normally. Therefore, it can be speculated that phagocytosis of PAM is completely blocked when PAM are infected with an moi of 100 of macrophage-grown PRRSV (Lena).

From these experiments we conclude that the effect of European genotype PRRSV on phagocytosis is determined by the total number of internalized virions. We suggest that both infectious and non-infectious virus contribute to the effect of European genotype PRRSV on phagocytosis, since at the same moi, the amount of internalized virions seems to determine the reduction in phagocytosis.

### European genotype PRRSV does not need to be infectious to downregulate PAM phagocytic capacity

**Figure 2:**
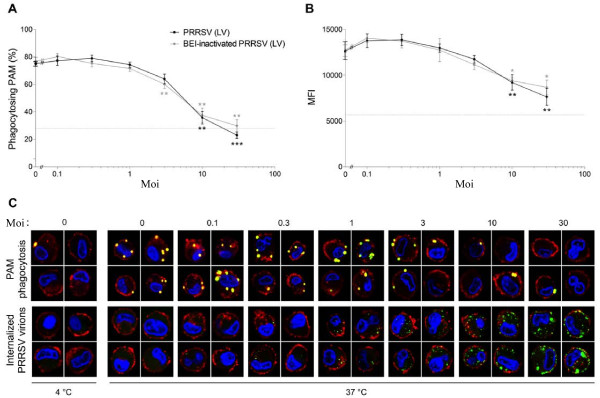
**European genotype PRRSV does not need to be infectious to downregulate PAM phagocytic capacity. (A–B)** Flow cytometric analysis of the role of PRRSV infectivity in the inhibitory effect of European genotype PRRSV on macrophage phagocytosis: The effect of Marc-145-grown infectious (Black circle) and BEI-inactivated (Grey circle) concentrated PRRSV (LV) on phagocytosis of beads by viable macrophages, expressed as the percentage of viable cells that have beads associated with them **(A)** or the MFI per viable cell **(B)**, which is a measure for the amount of beads per cell. PAM were inoculated with infectious or inactivated PRRSV (LV) at different moi as indicated and further incubated at 37°C for 1 h, after which a phagocytosis assay was performed. Mock infected cells were included as a control. Also, a phagocytosis assay was performed at 4°C (dotted line) after mock infection, to assess the percentage of macrophages that have beads bound to their surface and their MFI. Data represent the mean ± SEM of 4 independent experiments. *p < 0.05; ** *p* < 0.01; *** *p* < 0.001; **(C)** Confocal microscopical representation of the inhibitory effect of European genotype PRRSV on macrophage phagocytosis: the effect of Marc-145-grown, concentrated PRRSV (LV) on phagocytosis of beads by viable macrophages (performed as described above). An immunofluorescence staining was performed following the phagocytosis assay. Mock infected cells were included as a control and a phagocytosis assay was performed at 4°C after mock infection. For each moi, 4 single confocal z-sections throughout the middle of the cell are given, representative of each situation. Yellow-green fluorescent beads were used, nuclei are stained with Hoechst 33342 (blue), PRRSV virions are shown in green and cortical actin is visualized using TexasRed-X phalloidin. Upper panel: PAM phagocytosis of beads; Lower panel: internalized PRRSV particles.

Since the number of internalized virions appears to correlate with reduction in phagocytosis, it was investigated whether infectious PRRSV virions are needed for the inhibitory effect of European genotype PRRSV on PAM phagocytosis. Concentrated Marc-145-propagated PRRSV (LV) was BEI-inactivated, obtaining PRRSV virions that are able to bind and enter PAM similar to non-inactivated virions [29], without causing a productive infection. PAM were inoculated with either infectious or BEI-inactivated virus at different moi and further incubated at 37°C for 1 h, after which the effect on phagocytosis was studied (Figure 2). From our results, it is clear that BEI-inactivated PRRSV (LV) caused a similar reduction in phagocytosis as non-inactivated, infectious PRRSV (LV). In both cases, the percentage PAM phagocytosing beads (Figure 2A) and the number of beads phagocytosed per PAM (Figure 2B) was reduced starting from an moi of 3 and was further reduced when higher moi were administered. At an moi of 30, the percentage PAM phagocytosing beads was reduced down to the percentage observed when the assay was performed at 4°C (27.9 ± 0.6%), when active intracellular uptake is blocked, yet passive surface adsorption of beads proceeds normally. At this point, the number of beads per PAM was a little higher than at 4°C, as observed by MFI. This suggests that the phagocytic capacity of PAM is completely blocked at this point, even though more beads are bound at the cell surface. Similar results were obtained when UV-inactivated virus was used (data not shown). Hence, these findings show that the virus does not need to be infectious to cause a reduction in phagocytic capacity of PAM and it can therefore be assumed that binding and internalization of European genotype PRRSV virions into PAM is sufficient to reduce phagocytosis, without the need for viral replication. Figure 2c shows representative images which visualize that when a higher moi is administered, more viral particles have entered the cells and phagocytosis decreases.

### Ligand binding to sialoadhesin, but not CD163, downregulates phagocytosis

**Figure 3:**
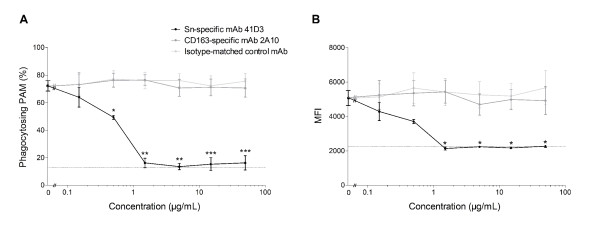
**Antibody binding to sialoadhesin, but not CD163, downregulates phagocytosis.** Flow cytometric analysis of the effect of Sn- or CD163-specific antibodies on macrophage phagocytosis. The effect of the Sn-specific mAb 41D3 (Black circle), the CD163-specific mAb 2A10 (Dark grey circle) or an isotype-matched control mAb 13D12 (Light grey circle) on phagocytosis of beads by viable macrophages, expressed as the percentage of viable cells that have beads associated with them **(A)** or the MFI per viable cell **(B)**, which is a measure for the amount of beads per cell. PAM were incubated with the indicated amount of mAb for 1 h, after which a phagocytosis assay was performed. As a control, non-treated PAM were included in the study. In addition, a phagocytosis assay was performed at 4°C (dotted line) with non-treated cells, to assess the percentage of macrophages that have beads bound to their surface and their MFI. Data represent the mean ± SEM of 3 independent experiments. *p < 0.05; **p < 0.01; ***p < 0.001.

Since inactivated virus also downregulates phagocytosis, the process causing this downregulation is not a replicative viral process, and probably results from a cellular process initiated upon interaction of the virus with the cell. Therefore we investigated whether the downregulation of phagocytosis could be explained by the interaction of the virus with its entry mediators Sn and/or CD163. To do so, another phagocytosis assay was performed. PAM were incubated with different concentrations of an Sn-specific mAb, a CD163-specific mAb or an isotype-matched control mAb for 1 h, after which the effect on phagocytosis was studied (Figure 3). Incubation of PAM with the Sn-specific mAb caused a downregulation of phagocytosis, starting from the lowest dose tested, affecting both the percentage of macrophages phagocytosing beads (Figure 3a) and the number of beads taken up per PAM (Figure 3b). PAM phagocytic capacity was further reduced when higher doses of the Sn-specific mAb were administered, up to 1.5 μg/mL, at which point phagocytosis was reduced down to the level observed when the assay was performed at 4°C, for both the percentage of PAM phagocytosing beads and the number of beads taken up per PAM. Further increasing the antibody dose did not further reduce the phagocytic capacity of PAM. This suggests that the phagocytic capacity of PAM is completely blocked when 1.5 μg/mL of the Sn-specific mAb is administered. Interestingly, the CD163-specific mAb 2A10 had no effect on phagocytosis, even at concentrations 30 times higher than the dose at which the Sn-specific mAb showed a maximum effect on phagocytosis. Similarly, experiments performed with a polyclonal antibody against CD163 also showed no effect on PAM phagocytosis (data not shown). In both cases, the effect of the CD163-specific antibodies was comparable to the effect of the isotype-matched control mAb treated or non-treated cells. Our data show that binding of the Sn-specific mAb to its receptor Sn leads to a similar inhibitory effect on PAM phagocytosis as observed after PAM inoculation with European genotype PRRSV, which suggests the involvement of Sn in the previously observed downregulation of phagocytosis by European genotype PRRSV. Our findings indicate that the observed downregulation of phagocytosis upon European genotype PRRSV inoculation is due to the interaction of the virus with its receptor Sn, but not CD163.

## Discussion

The present study shows that interaction of European genotype PRRSV with macrophages causes a clear reduction in macrophage phagocytic capacity, reducing both the number of macrophages phagocytosing beads and the number of beads taken up per cell. Using inactivated PRRSV (LV), we show that the observed downregulation of phagocytosis results from binding of PRRSV virions and their subsequent internalization, without the need for viral replication. This is supported by the fact that a higher number of internalized virions corresponds to a higher decrease in phagocytic capacity, both for Marc-145-grown and macrophage-grown virus. Finally, we suggest that the extracellular interaction of European genotype PRRSV with its receptor Sn, but not CD163, inhibits primary alveolar macrophage phagocytosis at the early stage of virus entry.

The fact that phagocytosis is inhibited 1 h after virus inoculation further supports our reasoning that the reduced phagocytic capacity is not merely due to virus-induced macrophage death, as often stated, and that the interaction of the PRRSV virion with its target cell is sufficient to reduce PAM phagocytosis. From previous research it is know that PRRSV has a replication cycle of approximately 12 h, after which the virus is released from the cell, thereby killing its target cell [37]. Moreover, it was shown that early in infection, PRRSV promotes anti-apoptotic pathways in PAM, up to 8 h post infection. Thus, at 1 h post infection, no virus-induced cell death occurs and therefore the decrease in phagocytosis cannot be explained by virus-induced cell-death.

It is known that 1 h post infection, internalized virions can still be observed, whereas 5 h post infection, virions can no longer be detected [38], which indicates that all virions are uncoated. Starting from 6 h post infection, newly synthesized structural proteins can be detected [39]. Consequently, this supports our finding that entry of the virus into the cell, but not production of progeny virus, causes the inhibition of phagocytosis.

Our study shows that a 1 h incubation of PAM with an Sn-specific mAb results in a decreased phagocytic capacity of PAM, whereas incubation with a CD163-specific mAb or pAb has no effect. Both Sn and CD163 are present at the cell surface and therefore available for antibody-binding. In addition, it was previously shown that incubation of PAM with any of these antibodies blocks infection of PRRSV, indicating that they can prevent interaction of the virus with their respective receptors ([28,30]; H. Van Gorp, unpublished data). Moreover, at the highest administered dose, still no effect on PAM phagocytic capacity of the CD163-specific antibodies was observed. This concentration was 33 times higher than the dose at which phagocytosis was completely blocked with the Sn-specific mAb. Therefore, we conclude that the interaction of European genotype PRRSV with its receptor Sn, but not CD163, inhibits primary alveolar macrophage phagocytosis.

Recent studies investigating the functional role of porcine Sn (pSn) have demonstrated that pSn is an endocytic receptor that mediates endocytosis of PRRSV or antibodies upon binding [26,31]. pSn was also shown to induce signalling [40] and reduce phagocytosis [27] in PAM upon antibody binding. Therefore, it was suggested that antibody binding to pSn activates certain molecular networks, which results in the downregulation of pathways involved in phagocytosis. This might also explain how the extracellular interaction of European genotype PRRSV with Sn downregulates phagocytosis. The results obtained in our study indicate that a certain number of virions need to interact with Sn before a certain ligand-receptor signalling threshold is reached and PAM get activated. In this scenario, the interaction of the virion with Sn serves as a trigger for further signalling, leading to a downregulation of PAM phagocytic capacity. Once this threshold is reached, administering higher moi, and therefore higher numbers of virions, results in a further downregulation of phagocytosis. Ligand-receptor signalling thresholds have been described for different viral ligand-receptor interactions, e.g. the interaction of human immunodeficiency virus (HIV) envelope glycoprotein (Env) with coreceptor CXCR4 [41] and of viral RNA with TLR7 [42], which must be reached to trigger sufficient activation of a receptor and subsequent signalling. Moreover, our results show that the phagocytic capacity of PAM can be blocked completely when a sufficiently high antibody dose or number of European genotype PRRSV particles (an moi of 30 of Marc-145-grown PRRSV (LV) or an moi of 100 of macrophage-grown PRRSV (Lena)) is administered. This suggests that once Sn is triggered sufficiently through ligand binding, whether it is an antibody or European genotype PRRSV virions, phagocytosis is maximally inhibited.

Both for human and mouse Sn it is suggested that alternative splicing of the Sn gene produces a transcript variant encoding an isoform that is soluble rather than membrane-bound [43]. It can be argued that these variants have no significant role in the downregulation of phagocytosis, since these variants are either excreted or present intracellularly in a non-membrane bound form, whereas surface-expressed Sn is needed to internalize PRRSV [31], subsequently downregulating phagocytosis. Moreover, no porcine splice variants have been reported, and no soluble Sn variants have so far been observed in vivo.

Interestingly, at an moi of 1, all macrophages have internalized several PRRSV virions, indicating that many non-infectious virions are present. This can be due to storage and thawing of the virus, but it can also be a direct result from the high mutation frequency reported for PRRSV [44,45]. If these mutations occur at crucial sites in the genome, the progeny virus will still be able to enter its target cell, but will have lost its ability to replicate upon uncoating and genome release. This observation is of importance if the observed inhibition of phagocytosis upon European genotype PRRSV infection also occurs in vivo, since non-infectious progeny virus is also present in the lungs of pigs, and can contribute to the downregulation of PAM phagocytic capacity.

Several other viruses have been shown to affect phagocytosis. Respiratory syncytial virus and secondary dengue virus infections have been shown to increase macrophage phagocytosis [46,47]. On the other hand, HIV, pseudorabies virus and influenza virus [48-50] infections are known to suppress phagocytosis, and in all cases this suppression is linked to an increased incidence of secondary infections. The way in which these viruses downregulate phagocytosis differs for each virus. For HIV for instance, one of the mechanisms causing the decreased phagocytosis in neutrophils and monocytes/macrophages is through the interaction of the cell with certain viral proteins, such as gp 120, p24 and Nef. It is difficult to comment on the relative moi at which phagocytosis is affected in these studies versus the moi at which European genotype PRRSV downregulates PAM phagocytosis. This is either due to different definitions of moi used in each study, such as egg infectious dose (EID_50_) or plaque forming units (PFU), or because persistently infected cells or cells isolated from infected individuals or animals were used and no moi was determined. It could be argued that the observed downregulation of PAM phagocytosis in our study occurs at relatively high moi, however, it is difficult to assess which moi are reached in the lungs of pigs in vivo. For PRRSV (Lena), titers of up to 10^7.8^ have been reported in BAL (bronchoalveolar lavage) fluids [13], corresponding to an moi of 63, and it can be argued that the effective titers in the lungs will be even higher, due to freeze/thawing and storage procedures.

PRRSV is a major player in porcine respiratory disease complex (PRDC) (reviewed by [24,51]). Field reports and clinical evidence demonstrate an increased incidence and severity of secondary infections associated with primary PRRSV infections. Strikingly, the direct effect of the virus on macrophage phagocytosis during the early stages of infection is poorly studied. Whereas some authors report that PRRSV infection has no effect on the uptake of *Staphylococcus aureus*[52] and *Escherichia coli*[53], other authors report an impaired phagocytic capacity against *Candida albicans*[54], *Salmonella typhimurium*[55], and *Haemophilus parasuis*[56], which supports our findings. Importantly, all of these studies showed, like we did, that virus-induced cell death was not involved. One of the factors influencing the outcome of these experiments is the secondary pathogen, and specific isolate, used. Each pathogen interacts with PAM in a different way and each secondary infection might have an additional immunomodulatory effect on PAM. Therefore, in our study, polystyrene beads were used as a reproducible, inert model to study the effect of European genotype PRRSV on PAM phagocytosis. Additionally, different PRRSV isolates might influence the outcome of each experiment, which was not the case for the two isolates used in this study. Yet, preliminary results indicate possible divergent effects of North American genotype PRRSV isolates on PAM phagocytosis (data not shown), which we are investigating in an ongoing study.

In a previous study, Van Gucht et al. [57] showed that infection with PRRSV sensitizes the lungs for production of proinflammatory cytokines upon exposure to lipopolysaccharides (LPS) and demonstrated that overproduction of these cytokines led to respiratory disease. If the observed inhibition of phagocytosis upon European genotype PRRSV infection also occurs in vivo, higher amounts of LPS will be present in the lungs after infection, since removal of secondary or opportunistic bacteria, and thus bacterial endotoxins, from the alveolar space will be affected. This could lead to a further exacerbation of cytokine production and more severe respiratory disease. This might also explain why an uncomplicated PRRSV infection often fails to induce overt respiratory disease [58], although PRRSV is a primary agent in PRDC.

To our knowledge, the present study is the first to show a direct effect of European genotype PRRSV on PAM phagocytic capacity in vitro, linking it to the interaction of the virus with its target cell, and more specific with its attachment and internalization receptor Sn. If similar events occur in vivo, this contributes to the reported increase and severity of secondary bacterial infections following European genotype PRRSV infection and the prevalence of European genotype PRRSV in porcine respiratory disease complex (PRDC).

## Abbreviations

BEI: Binary ethylenimine; HIV: Human immunodeficiency virus; LPS: Lipopolysaccharide; LV: Lelystad virus; MFI: Median fluorescence intensity; moi: multiplicity of infection; PAM: Primary alveolar macrophages; PRDC: Porcine respiratory disease complex; PRRS: Porcine reproductive and respiratory syndrome; PRRSV: Porcine reproductive and respiratory syndrome virus; pSn: porcine Sn; Sn: Sialoadhesin; UV: Ultraviolet.

## Competing interests

The authors declare that they have no competing interests.

## Authors’ contributions

MDB, HVG, PLD and HJN designed the study. MDB performed research, analyzed and interpreted data and wrote the manuscript. HVG, PLD and HJN contributed to the editing of the manuscript. PLD and HJN contributed important intellectual output and the supervision for the study. All authors read and approved the final manuscript.

## References

[B1] Collins JE Benfield DA Christianson WT Harris L Hennings JC Shaw DP Goyal SM McCullough S Morrison RB Joo HS Gorcyca D Chladek D Isolation of swine infertility and respiratory syndrome virus (Isolate ATCC VR-2332) in North-America and experimental reproduction of the disease in gnotobiotic pigs J Vet Diagn Invest 1992 4 117 126 10.1177/104063879200400201 1616975

[B2] Wensvoort G Terpstra C Pol JMA Terlaak EA Bloemraad M Dekluyver EP Kragten C Vanbuiten L Denbesten A Wagenaar F Broekhuijsen JM Moonen PLJM Zetstra T de Boer EA Tibben HJ de Jong MF van’t Veld P Groenland GJR van Gennep JA Voets MTh Verheijden JHM Braamskamp J Mystery swine disease in the Netherlands: the isolation of Lelystad virus Vet Q 1991 13 121 130 10.1080/01652176.1991.9694296 1835211

[B3] Holtkamp D Assessment of the economic impact of porcine reproductive and respiratory syndrome virus on U.S. pork producers 2011 International PRRS Symposium, Chicago, IL, U.S.A 86

[B4] Lewis CRG Ait-Ali T Clapperton M Archibald AL Bishop S Genetic perspectives on host responses to porcine reproductive and respiratory syndrome (PRRS) Viral Immunol 2007 20 343 357 10.1089/vim.2007.0024 17931105

[B5] Cavanagh D Nidovirales: a new order comprising Coronaviridae and Arteriviridae Arch Virol 1997 142 629 633 9349308

[B6] Meng XJ Paul PS Halbur PG Lum MA Phylogenetic analyses of the putative M(ORF-6)-gene and N(ORF-7)-gene of porcine reproductive and respiratory syndrome virus (PRRSV): implication for the existence of 2 genotypes of PRRSV in the USA and Europe Arch Virol 1995 140 745 755 10.1007/BF01309962 7794115 PMC7086766

[B7] Nelsen CJ Murtaugh MP Faaberg KS Porcine reproductive and respiratory syndrome virus comparison: divergent evolution on two continents J Virol 1999 73 270 280 9847330 10.1128/jvi.73.1.270-280.1999 PMC103831

[B8] Goldberg TL Lowe JF Milburn SM Firkins LD Quasispecies variation of porcine reproductive and respiratory syndrome virus during natural infection Virology 2003 317 197 207 10.1016/j.virol.2003.07.009 14698660

[B9] Meng XJ Heterogeneity of porcine reproductive and respiratory syndrome virus: implications for current vaccine efficacy and future vaccine development Vet Microbiol 2000 74 309 329 10.1016/S0378-1135(00)00196-6 10831854 PMC7117501

[B10] Shi M Lam TT-Y Hon C-C Hui RK-H Faaberg KS Wennblom T Murtaugh MP Stadejek T Leung FC-C Molecular epidemiology of PRRSV: a phylogenetic perspective Virus Res 2010 154 7 17 10.1016/j.virusres.2010.08.014 20837072

[B11] Stadejek T Oleksiewicz MB Scherbakov AV Timina AM Krabbe JS Chabros K Potapchuk D Definition of subtypes in the European genotype of porcine reproductive and respiratory syndrome virus: nucleocapsid characteristics and geographical distribution in Europe Arch Virol 2008 153 1479 1488 10.1007/s00705-008-0146-2 18592131

[B12] Zhou L Yang HC Porcine reproductive and respiratory syndrome in China Virus Res 2010 154 31 37 10.1016/j.virusres.2010.07.016 20659506

[B13] Karniychuk UU Geldhof M Vanhee M Van Doorsselaere J Saveleva TA Nauwynck HJ Pathogenesis and antigenic characterization of a new East European subtype 3 porcine reproductive and respiratory syndrome virus isolate BMC Vet Res 2010 6 30 10.1186/1746-6148-6-30 20525333 PMC2898778

[B14] Duan X Nauwynck HJ Pensaert MB Virus quantification and identification of cellular targets in the lungs and lymphoid tissues of pigs at different time intervals after inoculation with porcine reproductive and respiratory syndrome virus (PRRSV) Vet Microbiol 1997 56 9 19 10.1016/S0378-1135(96)01347-8 9228678

[B15] Duan X Nauwynck HJ Pensaert MB Effects of origin and state of differentiation and activation of monocytes/macrophages on their susceptibility to porcine reproductive and respiratory syndrome virus (PRRSV) Arch Virol 1997 142 2483 2497 10.1007/s007050050256 9672608 PMC7086874

[B16] Teifke JP Dauber M Fichtner D Lenk M Polster U Weiland E Beyer J Detection of European porcine reproductive and respiratory syndrome virus in porcine alveolar macrophages by two-colour immunofluorescence and in-situ hybridization-immunohistochemistry double labelling J Comp Pathol 2001 124 238 245 10.1053/jcpa.2000.0458 11437499

[B17] Choi C Chae C Expression of tumour necrosis factor-alpha is associated with apoptosis in lungs of pigs experimentally infected with porcine reproductive and respiratory syndrome virus Res Vet Sci 2002 72 45 49 12002637 10.1053/rvsc.2001.0519

[B18] Labarque G Van Gucht S Nauwynck H Van Reeth K Pensaert M Apoptosis in the lungs of pigs infected with porcine reproductive and respiratory syndrome virus and associations with the production of apoptogenic cytokines Vet Res 2003 34 249 260 10.1051/vetres:2003001 12791235

[B19] Sirinarumitr T Zhang YJ Kluge JP Halbur PG Paul PS A pneumo-virulent United States isolate of porcine reproductive and respiratory syndrome virus induces apoptosis in bystander cells both in vitro and in vivo J Gen Virol 1998 79 2989 2995 9880013 10.1099/0022-1317-79-12-2989

[B20] Sur JH Doster AR Osorio FA Apoptosis induced in vivo during acute infection by porcine reproductive and respiratory syndrome virus Vet Pathol 1998 35 506 514 10.1177/030098589803500605 9823592

[B21] Labarque GG Nauwynck HJ Van Reeth K Pensaert MB Effect of cellular changes and onset of humoral immunity on the replication of porcine reproductive and respiratory syndrome virus in the lungs of pigs J Gen Virol 2000 81 1327 1334 10769076 10.1099/0022-1317-81-5-1327

[B22] Van Gucht S Van Reeth K Nauwynck H Pensaert M Porcine reproductive and respiratory syndrome virus infection increases CD14 expression and lipopolysaccharide-binding protein in the lungs of pigs Viral Immunol 2005 18 116 126 10.1089/vim.2005.18.116 15802956

[B23] Schneberger D Aharonson-Raz K Singh B Monocyte and macrophage heterogeneity and Toll-like receptors in the lung Cell Tissue Res 2011 343 97 106 10.1007/s00441-010-1032-2 20824285

[B24] Brockmeier SL Halbur PG Thacker EL Brogden KA, Guthmiller JM Porcine Respiratory Disease Complex Polymicrobial Diseases 2002 ASM Press, Washington (DC) 231 258

[B25] Van Breedam W Delputte PL Van Gorp H Misinzo G Vanderheijden N Duan XB Nauwynck HJ Porcine reproductive and respiratory syndrome virus entry into the porcine macrophage J Gen Virol 2010 91 1659 1667 10.1099/vir.0.020503-0 20410315

[B26] Delputte PL Van Gorp H Favoreel HW Hoebeke I Delrue I Dewerchin H Verdonck F Verhasselt B Cox E Nauwynck HJ Porcine sialoadhesin (CD169/Siglec-1) is an endocytic receptor that allows targeted delivery of toxins and antigens to macrophages PloS One 2011 6 e16827 10.1371/journal.pone.0016827 21359217 PMC3040196

[B27] De Baere MI Van Gorp H Nauwynck HJ Delputte PL Antibody binding to porcine sialoadhesin reduces phagocytic capacity without affecting other macrophage effector functions Cell Immunol 2011 271 462 473 10.1016/j.cellimm.2011.08.016 21944562

[B28] Van Gorp H Van Breedam W Delputte PL Nauwynck HJ Sialoadhesin and CD163 join forces during entry of the porcine reproductive and respiratory syndrome virus J Gen Virol 2008 89 2943 2953 10.1099/vir.0.2008/005009-0 19008379

[B29] Vanhee M Delputte PL Delrue I Geldhof MF Nauwynck HJ Development of an experimental inactivated PRRSV vaccine that induces virus-neutralizing antibodies Vet Res 2009 40 63 10.1051/vetres/2009046 19674539

[B30] Duan XB Nauwynck HJ Favoreel HW Pensaert MB Identification of a putative receptor for porcine reproductive and respiratory syndrome virus on porcine alveolar macrophages J Virol 1998 72 4520 4523 9557752 10.1128/jvi.72.5.4520-4523.1998 PMC109698

[B31] Vanderheijden N Delputte PL Favoreel HW Vandekerckhove J Van Damme J van Woensel PA Nauwynck HJ Involvement of sialoadhesin in entry of porcine reproductive and respiratory syndrome virus into porcine alveolar macrophages J Virol 2003 77 8207 8215 10.1128/JVI.77.15.8207-8215.2003 12857889 PMC165228

[B32] Bullido R delMoral MG Alonso F Ezquerra A Zapata A Sanchez C Ortino E Alvarez B Dominquez J Monoclonal antibodies specific for porcine monocytes/macrophages: Macrophage heterogeneity in the pig evidenced by the expression of surface antigens Tissue Antigens 1997 49 403 413 10.1111/j.1399-0039.1997.tb02769.x 9151393

[B33] Sanchez C Domenech N Vazquez J Alonso F Ezquerra A Dominguez J The porcine 2A10 antigen is homologous to human CD163 and related to macrophage differentiation J Immunol 1999 162 5230 5237 10227997

[B34] Nauwynck HJ Pensaert MB Effect of specific antibodies on the cell-associated spread of pseudorabies virus in monolayers of different cell types Arch Virol 1995 140 1137 1146 10.1007/BF01315422 7611884

[B35] Van Breedam W Sarah C Vanhee M Gagnon CA Rodriguez-Gomez IM Geldhof M Verbeeck M Van Doorsselaere J Karniychuk U Nauwynck HJ Porcine reproductive and respiratory syndrome virus (PRRSV)-specific mAbs: supporting diagnostics and providing new insights into the antigenic properties of the virus Vet Immunol Immunopathol 2011 141 246 257 10.1016/j.vetimm.2011.03.008 21470695

[B36] Delputte PL Meerts P Costers S Nauwynck HJ Effect of virus-specific antibodies on attachment, internalization and infection of porcine reproductive and respiratory syndrome virus in primary macrophages Vet Immunol Immunopathol 2004 102 179 188 10.1016/j.vetimm.2004.09.007 15507304

[B37] Costers S Lefebvre DJ Delputte PL Nauwynck HJ Porcine reproductive and respiratory syndrome virus modulates apoptosis during replication in alveolar macrophages Arch Virol 2008 153 1453 1465 10.1007/s00705-008-0135-5 18563285

[B38] Misinzo GM Delputte PL Nauwynck HJ Involvement of proteases in porcine reproductive and respiratory syndrome virus uncoating upon internalization in primary macrophages Vet Res 2008 39 35 10.1051/vetres:2008012 18651989

[B39] Costers S Delputte PL Nauwynck HJ Porcine reproductive and respiratory syndrome virus-infected alveolar macrophages contain no detectable levels of viral proteins in their plasma membrane and are protected against antibody-dependent, complement-mediated cell lysis J Gen Virol 2006 87 2341 2351 10.1099/vir.0.81808-0 16847130

[B40] Genini S Malinverni R Delputte PL Fiorentini S Stella A Botti S Nauwynck HJ Giuffra E Gene expression profiling of porcine alveolar macrophages after antibody-mediated cross-linking of sialoadhesin (Sn, Siglec-1) J Recept Signal Transduct Res 2008 28 185 243 10.1080/10799890802084226 18569525

[B41] Melar M Ott DE Hope TJ Physiological levels of virion-associated human immunodeficiency virus type 1 envelope induce coreceptor-dependent calcium flux J Virol 2007 81 1773 1785 10.1128/JVI.01316-06 17121788 PMC1797554

[B42] Wang JP Liu P Latz E Golenbock DT Finberg RW Libraty DH Flavivirus activation of plasmacytoid dendritic cells delineates key elements of TLR7 signaling beyond endosomal recognition J Immunol 2006 177 7114 7121 17082628 10.4049/jimmunol.177.10.7114

[B43] Crocker PR Mucklow S Bouckson V McWilliam A Willis AC Gordon S Milon G Kelm S Bradfield P Sialoadhesin, a macrophage sialic acid binding receptor for hematopoietic cells with 17 immunoglobulin-like domains Embo J 1994 13 4490 4503 7925291 10.1002/j.1460-2075.1994.tb06771.x PMC395382

[B44] Hanada K Suzuki Y Nakane T Hirose O Gojobori T The origin and evolution of porcine reproductive and respiratory syndrome viruses Mol Biol Evol 2005 22 1024 1031 10.1093/molbev/msi089 15659555 PMC7107557

[B45] Jenkins GM Rambaut A Pybus OG Holmes EC Rates of molecular evolution in RNA viruses: a quantitative phylogenetic analysis J Mol Evol 2002 54 156 165 10.1007/s00239-001-0064-3 11821909

[B46] Guerrero-Plata A Ortega E Gomez B Persistence of respiratory syncytial virus in macrophages alters phagocytosis and pro-inflammatory cytokine production Viral Immunol 2001 14 19 30 10.1089/08828240151061347 11270594

[B47] Honda S Saito M Dimaano EM Morales PA Alonzo MTG Suarez LAC Koike N Inoue S Kumatori A Matias RR Natividad FF Oishi K Increased phagocytosis of platelets from patients with secondary dengue virus infection by human macrophages Am J Trop Med Hyg 2009 80 841 845 19407135

[B48] Astry CL Jakab GJ Influenza virus-induced immune-complexes suppress alveolar macrophage phagocytosis J Virol 1984 50 287 292 6708169 10.1128/jvi.50.2.287-292.1984 PMC255619

[B49] Iglesias G Pijoan C Molitor T Interactions of pseudorabies virus with swine alveolar macrophages: effects of virus infection on cell functions J Leukocyte Biol 1989 45 410 415 2708911 10.1002/jlb.45.5.410 PMC7166681

[B50] Pugliese A Vidotto V Beltramo T Torre D Phagocytic activity in human immunodeficiency virus type 1 infection Clin Diagn Lab Immunol 2005 12 889 895 16085904 10.1128/CDLI.12.8.889-895.2005 PMC1182180

[B51] Opriessnig T Giménez-Lirola LG Halbur PG Polymicrobial respiratory disease in pigs Anim Health Res Rev 2011 12 133 148 10.1017/S1466252311000120 22152290

[B52] Thanawongnuwech R Thacker EL Halbur PG Effect of porcine reproductive and respiratory syndrome virus (PRRSV) (isolate ATCC VR-2385) infection on bactericidal activity of porcine pulmonary intravascular macrophages (PIMs): in vitro comparisons with pulmonary alveolar macrophages (PAMs) Vet Immunol Immunopathol 1997 59 323 335 10.1016/S0165-2427(97)00078-0 9477481

[B53] Oleksiewicz MB Nielsen J Effect of porcine reproductive and respiratory syndrome virus (PRRSV) on alveolar lung macrophage survival and function Vet Microbiol 1999 66 15 27 10.1016/S0378-1135(98)00309-5 10223319

[B54] Chiou MT Jeng CR Chueh LL Cheng CH Pang VF Effects of porcine reproductive and respiratory syndrome virus (isolate tw91) on porcine alveolar macrophages in vitro Vet Microbiol 2000 71 9 25 10.1016/S0378-1135(99)00159-5 10665530

[B55] Riber U Nielsen J Lind P In utero infection with PRRS virus modulates cellular functions of blood monocytes and alveolar lung macrophages in piglets Vet Immunol Immunopathol 2004 99 169 177 10.1016/j.vetimm.2004.02.008 15135983

[B56] Solano GI Bautista E Molitor TW Segales J Pijoan C Effect of porcine reproductive and respiratory syndrome virus infection on the clearance of Haemophilus parasuis by porcine alveolar macrophages Can J Vet Res 1998 62 251 256 9798089 PMC1189490

[B57] Van Gucht S Van Reeth K Pensaert M Interaction between porcine reproductive-respiratory syndrome virus and bacterial endotoxin in the lungs of pigs: potentiation of cytokine production and respiratory disease J Clin Microbiol 2003 41 960 966 10.1128/JCM.41.3.960-966.2003 12624016 PMC150282

[B58] Van Reeth K Labarque G Nauwynck H Pensaert M Differential production of proinflammatory cytokines in the pig lung during different respiratory virus infections: correlations with pathogenicity Res Vet Sci 1999 67 47 52 10.1053/rvsc.1998.0277 10425240 PMC7126504

[B59] UGent TechTransfer Website [www.techtransfer.ugent.be].

[B60] Flemish Institute for the Promotion of Innovation by Science and Technology Website [www.iwt.be].

